# A Super Energy Mitigation Nanostructure at High Impact Speed Based on Buckyball System

**DOI:** 10.1371/journal.pone.0064697

**Published:** 2013-05-28

**Authors:** Jun Xu, Yibing Li, Yong Xiang, Xi Chen

**Affiliations:** 1 Columbia Nanomechanics Research Center, Department of Earth and Environmental Engineering, Columbia University, New York, New York, United States of America; 2 State Key Laboratory of Automotive Safety & Energy, Department of Automotive Engineering, Tsinghua University, Beijing, China; 3 State Key Lab of Electronic Thin Films and Integrated Devices, School of Energy Science and Engineering, University of Electronic Science and Technology of China, Chengdu, Sichuan, China; 4 Department of Civil and Environmental Engineering, Hanyang University, Seoul, Korea; 5 International Center for Applied Mechanics, SV Lab, Xi'an Jiaotong University, Xi'an, China; UC Davis School of Medicine, United States of America

## Abstract

The energy mitigation properties of buckyballs are investigated using molecular dynamics (MD) simulations. A one dimensional buckyball long chain is employed as a unit cell of granular fullerene particles. Two types of buckyballs i.e. C_60_ and C_720_ with recoverable and non-recoverable behaviors are chosen respectively. For C_60_ whose deformation is relatively small, a dissipative contact model is proposed. Over 90% of the total impact energy is proven to be mitigated through interfacial reflection of wave propagation, the van der Waals interaction, covalent potential energy and atomistic kinetic energy evidenced by the decent force attenuation and elongation of transmitted impact. Further, the C_720_ system is found to outperform its C_60_ counterpart and is able to mitigate over 99% of the total kinetic energy by using a much shorter chain thanks to its non-recoverable deformation which enhances the four energy dissipation terms. Systematic studies are carried out to elucidate the effects of impactor speed and mass, as well as buckyball size and number on the system energy mitigation performance. This one dimensional buckyball system is especially helpful to deal with the impactor of high impact speed but small mass. The results may shed some lights on the research of high-efficiency energy mitigation material selections and structure designs.

## Introduction

Protection of materials and devices under high-speed impact, whose most critical task is energy mitigation and absorption [1]–[3], poses a major challenge in engineering. For ballistic loading, i.e. high impact speed with small impact mass, the force attenuation should be the priority [4] to effectively mitigate impact energy. Woven fabric composites [5]–[8], sandwich structure [9]–[11], metal foams [12]–[14] and nanomaterials [15]–[21] are widely used for energy mitigation upon high speed impact, which primarily consume the impact energy through widespread failure or extensive deformation.

Granular material arranging in a chain-like structure [22], [23] is attractive for force attenuation, and such a discrete system effectively responds to impact loading via stress wave propagation across various interfaces to reduce the transmitted force. Pioneering work on the characteristics of the solitary wave propagation in a homogeneous chain of metallic spheres based on the Hertz contact law was established by Nesterenko [24]. Since then, many contributions have been put forward to refine the chain system for outstanding energy damping ability, including the material and geometrical parameters [25], [26], arrangements [27], [28], and model parameterizations of different granular materials [29]–[31].

Recently, with the development of nanomaterial, carbon nanotubes (CNTs) [21], [32] have been one of the promising candidates for impact energy absorption thanks to its ultra-high modulus and strength [33]–[35]. Buckyballs, another branch of fullerene family, also have high potential for energy mitigation owing to their excellent mechanical properties and unique morphology [36], [37]. According to our previous work [20], [38], the progressive buckling and densification in response to impact loading, as well as the particular non-recoverable portraits of larger buckyballs, may help to dissipate and absorb intense stress waves. Thus, inspired by granular materials, it is envisioned that the stacking of nano-sized buckyballs could exhibit excellent energy mitigation capabilities.

In this paper, two representative buckyballs C_60_ and C_720_ stacked in one-dimensional chain-like system are chosen to study the mechanical behavior subject to high speed impact. For the small C_60_ buckyball chain, an analytical model based on the Hertz contact law is suggested by analogy to the fundamental Nesterenko's model. Molecular dynamics (MD) simulations are employed to study the transmitted force history and the peak force attenuation. Stress wave propagation characteristics are also investigated such that system effective response is evaluated. For the giant C_720_ buckyball chain, MD simulations are used to compute the contact forces on the impactor and receiver, as well as the stress wave propagation. Further, the effect of the impact mass and speed on the system performance is thoroughly studied to fully unveil the energy mitigation mechanism. Finally, buckyballs with various sizes are embedded into the chain system to explore the particle size effect on the energy dissipation ability.

## Computational Model and Method

Small and large buckyballs behave differently upon impact: the smaller ones are often resilient while the larger ones exhibit non-recovery phenomenon after unloading [38]. In this study, C_60_ and C_720_ are selected to represent “recoverable buckyball” and “non-recoverable buckyball” respectively. In continuum modeling, buckyballs are assumed to share the same effective Young's modulus *E* = 5 TPa and nominal wall thickness *t* = 0.66 nm [38]. The densities of C_720_ and C_60_ are 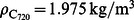 and 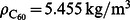 respectively. The other basic physical parameters of C_720_ and C_60_ are listed in Ref. [20].

**Figure 1:**
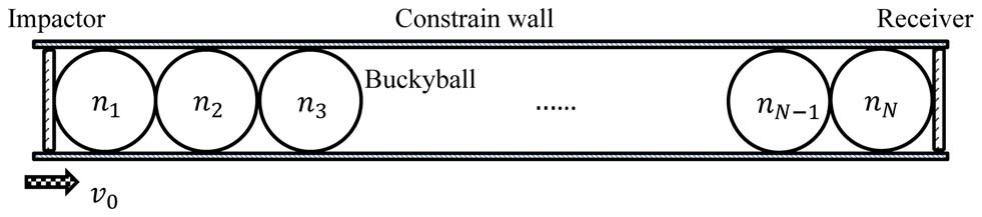
Illustration of one-dimensional buckyball chain setup as an impact protector.

To simulate a granular system, we assume the identical buckyballs are packed in a simple cubic manner such that the stress wave would be confined within one dimension (effects caused by different packaging arrangements have been discussed in Ref [38]). We have shown that the system deformation mode and the energy absorption/mitigation ability are independent of the arrangement number in both vertical and horizontal lineups in previous work [38]. In addition, preliminary simulation also reveals that system with multi-column stacking has no obvious difference in deformation behavior and unit energy absorption rate. Thus, by taking advantage of symmetry, a long chain of buckyball system is simulated. The “long chain” is set to be at least 20 times in length than its width, and a typical system contains 100 buckyballs. The computational cell is illustrated in Figure 1, where the buckyball system subjects to the impact of a rigid left plate with incident energy *E*_impactor_ and the impact speed is varied from 100 m/s to 1000 m/s which is conventionally considered as high impact speed domain, mainly aiming at the ballistic impact related problem. Mass changing falls into the domain where the maximum strain is large enough while the temperature rising of the buckyball caused by the kinetic energy is below 800 K when buckyball may remain stable. A rigid and fixed right plate serves as a receiver which would indicate the energy mitigation capability of the protective system (the buckyball chain is sandwiched between the plates). Force histories on the left and right plates are recorded.

A full atomistic description of the buckyball is used. MD simulation is performed based on LAMMPS (large-scale atomic/molecular massively parallel simulator) platform with the NVE ensemble (micro-canonical ensembles) [39] after running initial equilibrium. A pairwise Lennard-Jones (L-J) potential term is added to the buckyball potential to account for the steric and van der Waals carbon–carbon interactions 
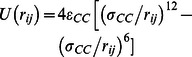
 where 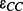 is the depth of the potential well between carbon-carbon atoms; 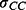 is the finite distance where the carbon-carbon potential is zero; 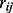 is the distance between the two carbon atoms. Here, L-J parameters for the carbon atoms of the buckyball are 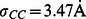 and 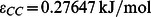 as used in the original parameterization of Girifalco [40] and Van der Waals interaction governs in the plate-buckyball interaction. Carbon atoms are employed to make both the impactor and receiver plates with varying masses in the following simulation to set various loading conditions (varying impactor mass) while the interactions between the plates and buckyballs remain as carbon-carbon ones. Details of the simulation methods are described elsewhere [38]. To simulate the long one dimensional chain, four L-J walls with the same parameters are set as four sides of the simulation box to provide necessary lateral constraints from simple cubic packing. A time integration step of 1 fs is used and periodical boundary conditions are applied in the *x*,*y* plane to eliminated the boundary effect.

## Representative Impact Behavior

### Dynamic response of C_60_ chain system

#### 1 Hertzian model

Interactions between particles in the one-dimensional chain system subject to contact loading may be treated based on the Hertz law [24]. Similar to granular particles, each C_60_ molecule in the chain system undergoes relatively small deformation without any buckling or bifurcation. In addition, the characteristic time 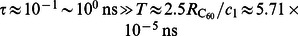 where 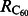 is the radius of C_60_ and 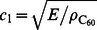 is the wave speed [24]. Therefore, the Hertz contact law still approximately holds for the dynamic response of C_60_ chain system.

Consider a one dimensional chain of *N* same C_60_s with mass 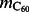, radius 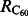 and Young's modulus 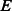 and Poisson's ratio 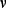 in contact without any precompression. The Hertzian contact law between neighboring buckyballs and could be expressed as [41]

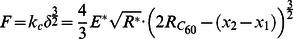
(1)where *F* is the contact force, 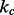 referring the elastic coefficient, 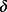 is deformation, *x*_2,_ *x*_1_ are coordinates of two neighboring buckyball centers (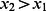); 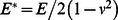, and 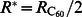 are the effective Young's modulus and effective radius respectively. By replacing the coordinate 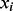 by the displacement 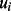 of the *i*th buckyball from its equilibrium position in the chain, the equation of motion for each buckyball may be further written as
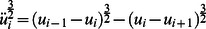
(2)This is widely used for granular materials.

#### 2 MD simulation of one-dimensional C_60_ chain

**Figure 2:**
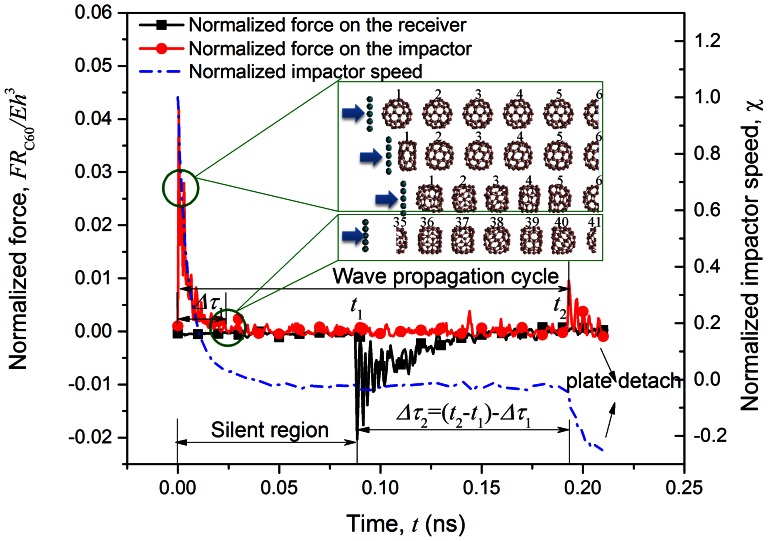
Normalized force time history and impactor velocity history of C_60_ chain containing 100 buckyballs, when the impact energy is 6.49 eV and impact speed is 500 m/s.

The forces on both impactor and receiver plates are normalized as 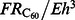, and the representative impact force attenuation for 100 C_60_ particles is shown in Figure 2 (where the positive value stands for compression force along the impact velocity direction). A sharp and narrow impact pulse is initiated once the top plate collides with the buckyball system and it drops to nearly zero at about 0.02 ns (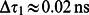), indicating that the compressive stress wave is traveling towards the receiver. The receiver does not experience any force until the stress wave arrives at 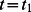 (shown in Figure 2); from which the average traveling speed of the stress wave is estimated as 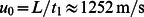 and thus the system equivalent modulus is 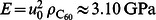 for the specific impact loading condition (impact energy of 6.49 eV and impact speed of 500 m/s). Once the stress wave reaches the receiver, it reflects back and if it successfully travels back to the impactor, a secondary impact impulse would form at 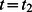 (shown in Figure 2) and thus causes the speed of the ricochet impactor increase again. The peak transmitted force on the receiver is about 42.27% of the original peak force on the impactor, after force attention of 100 C_60_ buckyballs. About 93.75% of the impactor kinetic energy (i.e. impact energy) is dissipated by the system, therefore, one may define the energy mitigation rate as 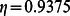. The effect of buckyball number on the energy mitigation rate is discussed later.

According to the force equilibrium and mass continuity, the stress of a particular mass point during the wave propagation is 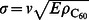 [42], and the relation between stress 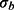 and 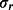 (at the interface of buckyball and the rigid plate respectively) may be expressed as [42]

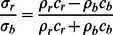
(3)where 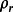 and 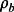 are densities and 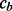 and 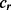 are wave speeds of the material on the two sides of the interface respectively. Similarly, the stress wave speed may be written as [42]

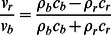
(4)Since the receiver is fixed as a rigid body in this study, 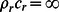, such that 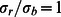 and 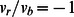 which means that the stress wave propagates back to impactor at the same speed. After the reflective wave travels through 100 C_60_ buckyballs, the magnitude of force on impactor reduces to 21.95% of the original force. On the other hand, the transmitted force pulse duration is about 5.4 times of that on the impactor, i.e. 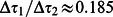, showing a prominent stress wave mitigation effect. The major energy mitigation effect results from the stress wave attenuation caused by the reflections among buckyball walls, similar as that found in previous research in granular system [22], [25], [29], [30], [43], as well as the van der Waals interactions between buckled layers and similar energy absorption mechanism revealed in carbon nanotubes in Ref. [17], [18], [44]. In addition, about 1.5% of the impact energy may be converted to the kinetic energy of the atoms within C_60_.

#### 3 Dissipative Hertzian model

**Figure 3:**
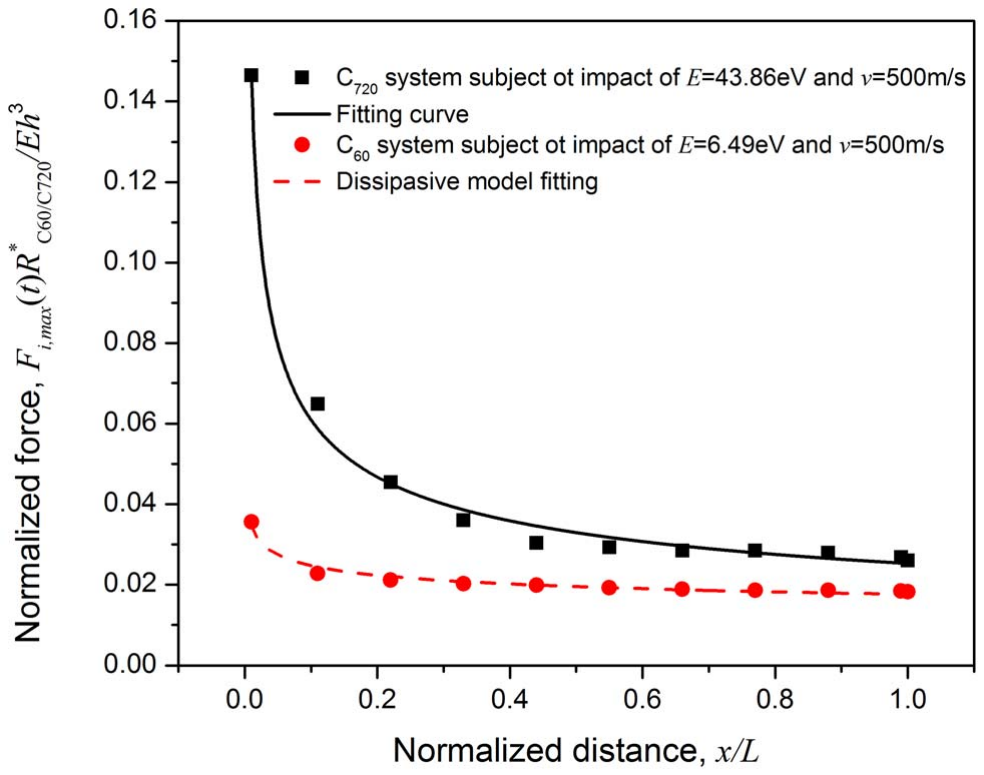
The normalized force distribution on selected buckyballs in C_60_ and C_720_ chain systems.

As the method adopted in Ref [45] to include the dissipation term to Eq. (2), from MD simulation, the following relationship can be fitted:
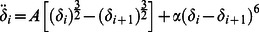
(5)where 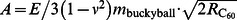, the second term implies dissipation which is fitted based on the force-displacement curve at large deformation in our previous study [20], [38] and its coefficient 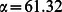 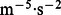. This relationship is valid for systems with large number of C_60_ buckyballs at all loading conditions as long as the Hertzian contact law holds. Figure 3 shows the maximum force on the *i*th ball, 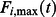, of the dissipative model (Eq. (5)), which is consistent with the MD results of C_60_ chains.

### Dynamic response of C_720_ chain system

**Figure 4:**
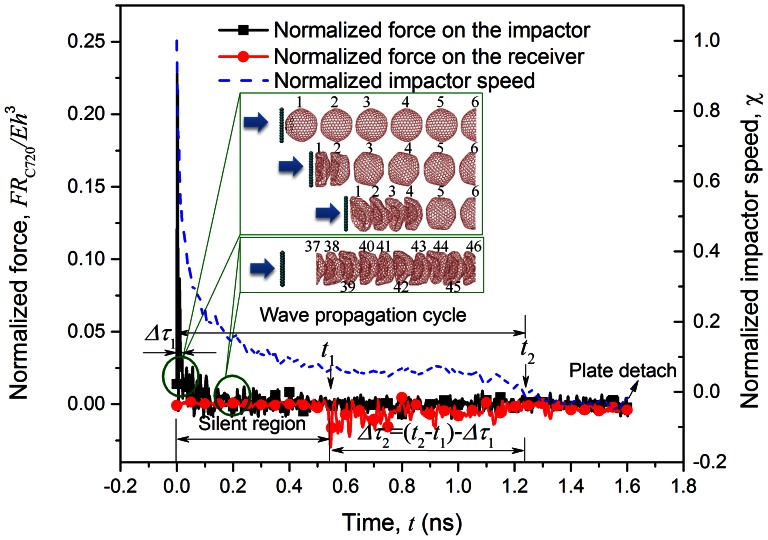
Normalized force history and impactor velocity history of C_720_ chain containing 100 buckyballs, when the impact energy is 6.49 eV and impact speed is 500 m/s.

The large non-recoverable deformation of C_720_ makes the Hertzian contact law invalid. The energy mitigation behaviors are investigated using MD simulations. Typical normalized force history curves of the impactor and receiver are shown in Figure 4, where 100 C_720_ are studied. In terms of stress wave traveling, its average speed is 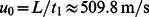, and thus the system equivalent Young's modulus is 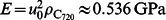, which means the C_720_ chain system is more “compliant” than C_60_. In our previous work, the “non-recovery” phenomenon is proven to be only strain determined, regardless of the impact mass and velocity [38]. During preliminary simulations, we also confirm that the “non-recovery” phenomenon in C_540_ is impact-condition independent.

From energy mitigation perspective, a very sharp initial impulse is attenuated to a much milder and longer impulse. The ratio of the peak magnitude and duration between the original and transmitted impulses are 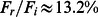 (where the subscript *r* and *i* refer to the receiver and impactor respectively) and 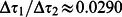. The force reduction and duration elongation are much higher than that in C_60_ chain system due to the buckled-through shape of C_720_ during impact. Therefore, van der Waals interactions between buckled and “stickered” layers may contribute more energy dissipation compared to its counterpart in C_60_ system due to the un-recoverable deformation. Also, with the buckled morphorlogy of C_720_, the covalent potential energy also increase via the consumption of external impact energy. Moreover, about 12% of the impact energy could be mitigated in the form of atom kinetic energy which also contributes the superiority of energy dissipation for C_720_ system. The power-law-like dissipative model for contact force attenuation 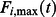 at various buckyballs still applies (see Fig. 3), indicating a fast force decay along the wave propagation direction. In the meantime, over 99% of the impact energy is mitigated to the kinetic energy and strain energy of buckyballs.

## Parametric Study and Discussions

**Figure 5:**
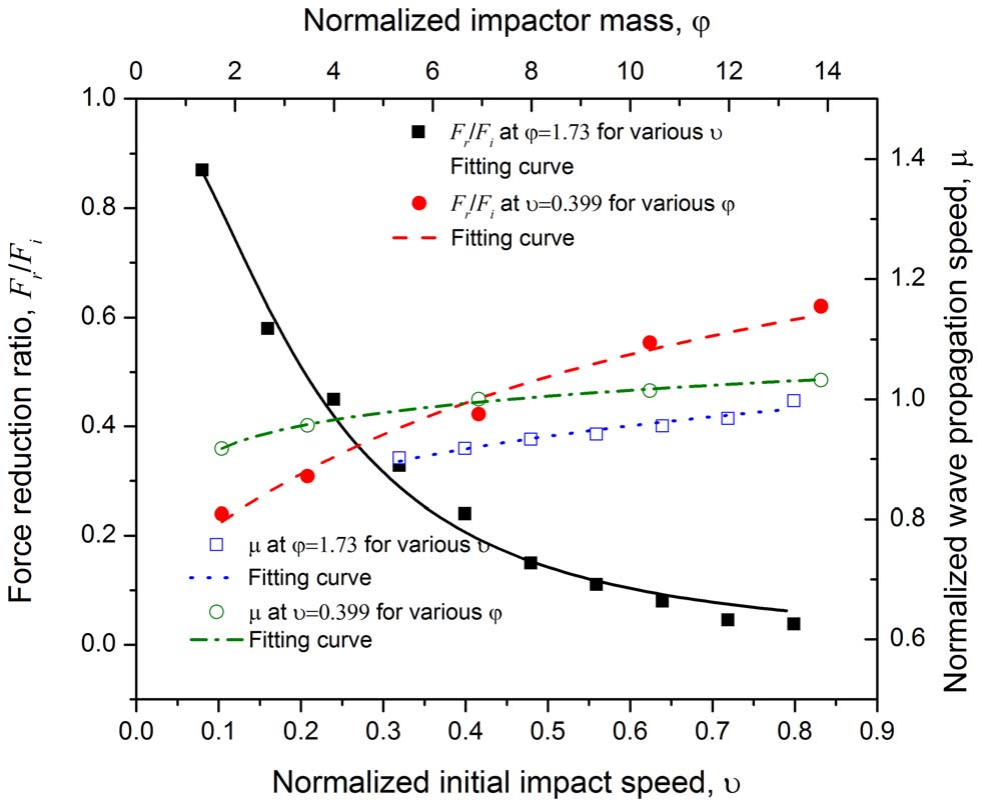
Force reduction ratio 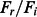 and normalized wave propagation speed 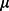 under various impact speeds (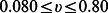) with fixed impact mass (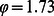), as well as various impact masses (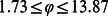) with fixed impact speed (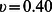) for C_60_ chain system containing 100 buckyballs. Nonlinear models are suggested to fit the computational data.

**Figure 6:**
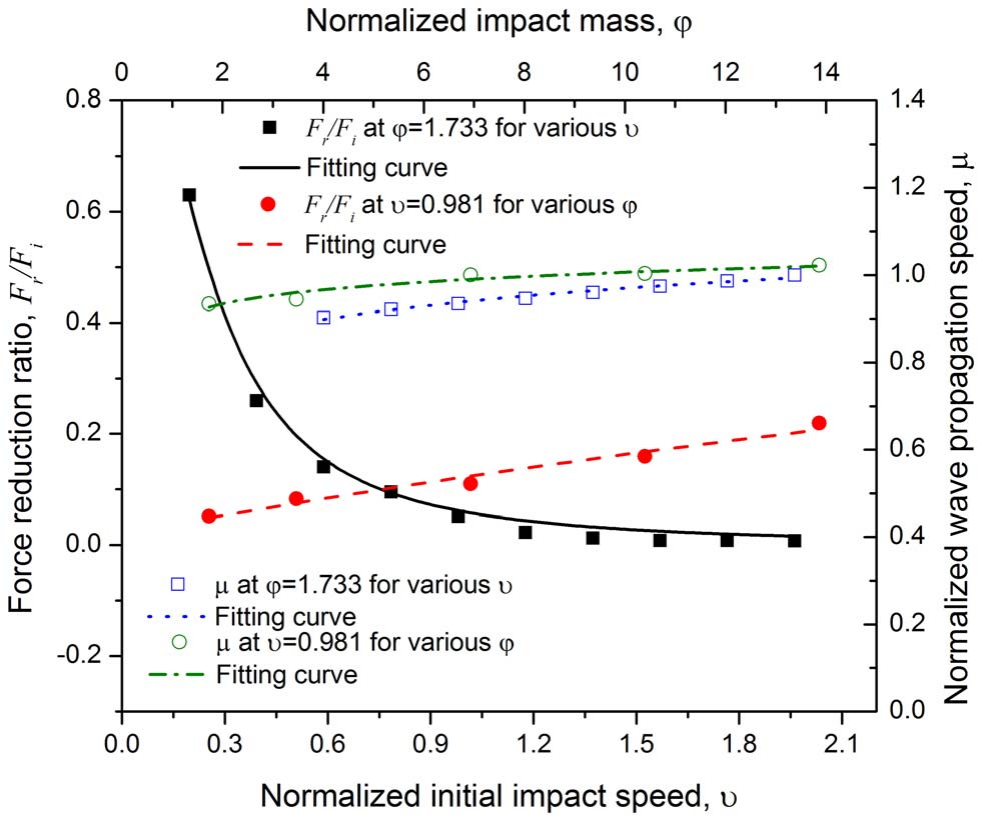
Force reduction ratio 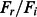 and normalized wave propagation speed 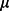 under various impact speeds (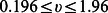) with fixed impact mass (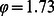), as well as various impact masses (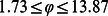) with fixed impact speed (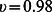) for C_720_ chain system containing 100 buckyballs. Nonlinear models are suggested to fit the computational data.

A parametric study is carried out where the impact speed is varied from 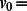
100 m/s to 1000 m/s, and the impact mass per carbon atom is varied at 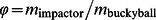 = 1.73 to 13.87 for both the C_60_ and C_720_ chains containing 100 buckyballs. The initial impact speed is normalized as 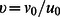 (where 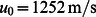 and 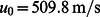 are used for C_60_ and C_720_ chains respectively); the stress wave propagation speed (calculated based on the time when the wave transmits through the chain, which is dependent on the number of buckyballs) is normalized as 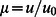. The corresponding fitting curves of the suggested models are also shown in Figures 5 and 6.

### Effects of initial impact speed and mass on C_60_ chain

#### 1 Force attenuation

The force reduction ratio 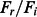 and normalized wave propagation speed 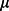 are two indices employed to evaluate the mitigation properties, shown in Figure 5. Following Reid and Peng [46], the enhanced dynamic stress 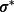 can be expressed as
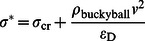
(6)where 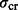 is the crushing strength of buckyball and the 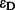 is the material strain attained behind the wave front. *v* is the particle velocity at a certain time *t*. By keeping the impact mass constant, the particle velocity, 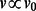 [46]. Assuming the contact area keeps a constant as 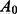, one may come to 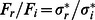. Thus, the force reduction ratio under a fixed impact mass (but different impact velocities) is
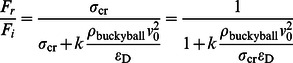
(7)where *k* is the linear coefficient between 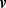 and 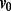. Alternatively, under this condition, we may fit the equation in the form of
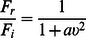
(8)where *a* is material-related parameter and from Figure 5 it yields 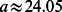 for the present system.

One may also regard the buckyball system as a non-linear spring damping system whose stiffness is only slightly affected by the mass of the impactor. Such a damping system reduces the force in the receiver by extending the functioning time over a longer time period. When the impact speed remains the same but impact mass is different, the following form is fitted to describe the force attenuation
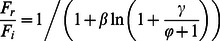
(9)where 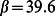 and 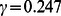 for the present system.

Eqs. (8) and (9) in together reveal that the one-dimensional C_60_ chain system has a better mitigation performance under the condition of higher impact speed with smaller mass, in terms of the force attenuation to alleviate the transmitted load on objects to be protected.

#### 2 System equivalent Young's modulus

The system equivalent Young's modulus may be characterized via the elongation of wave propagation speed. The mitigation behavior is still dominated by impact energy, which means that changing the impactor mass or speed may vary the mitigation performance. The ratio between dynamic stress 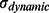 and static stress 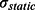 for rate-sensitive material may be expressed as [47]

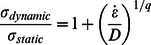
(10)where 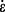 is the strain rate, *D* and *q* are constants for a particular material. With the relation between stress and Young's modulus as well as the strain rate and velocity, one may fit the normalized wave propagation speed with varying impact speed 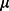 (yet same impact mass) in the form of
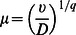
(11)where 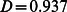 and 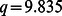. Combining the two equations above yields the relationship under various impact masses (but same impact speed):
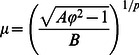
(12)where 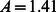, 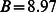 and 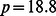 through the best fitting.

Note that when the number of buckyballs in the system increases, the effective system rigidity becomes smaller due to the longer stress wave transmission. Therefore, the fitted formula for calculating the stress wave speed and the corresponding equivalent rigidity are only valid for this specific system under subscribed loading conditions. However, these parametric values may become numerically convergent under certain impact mass once the number of buckyball reaches the threshold value which is discussed later.

### Effect of initial impact speed and mass on C_720_ chain

#### 1 Force attenuation

To evaluate the energy mitigation performance of C_720_ chain, the force reduction ratio 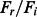 and normalized wave propagation speed 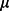 are employed in Figure 6. The 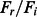 value reduces sharply in the relatively low impact speed domain and becomes stable once the impact speed exceeds over 500 m/s. Eq. (8) still applies with 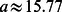 via best fitting.

Due to the non-recoverable deformation of C_720_, the impact mass poses stronger influence over the force reduction ratio because the larger mass makes the first few buckyballs easier to buckle. With the impactor mass increasing, the force reduction is also more prominent than that in C_60_ chain system. Similarly, Eq. (10) may be applied with 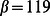 and 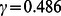.

#### 4.2.2 System equivalent Young's modulus

Similarly, we may also take the form of Eqs. (11) and (12) to describe the system equivalent rigidity based on stress wave propagation speed. With the impact speed increases, the average wave propagation speed also increases, leading to a much stiffer system in terms of rigidity. In Eq. (11), the fitting parameters are 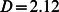 and 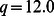 for the C_720_ buckyball system. By taking the derivative of Eq. (11), the variation rate in C_60_ is more prominent than that of C_720_, indicating that C_60_ exhibit even higher effective stiffness than C_720_ under very high impact speed situations. The fitting of Eq. (12) yields 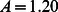, 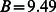 and 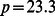 for the C_720_ chain system, indicating that the effect of impactor mass is less on C_720_ than that on C_60_ chain. Again, these fitted equations are only valid for the protective system with particular number of buckyball under the specific loading conditions. System rigidity would also alter accordingly if any of the corresponding factors change.

### Effect of buckyball size

**Figure 7:**
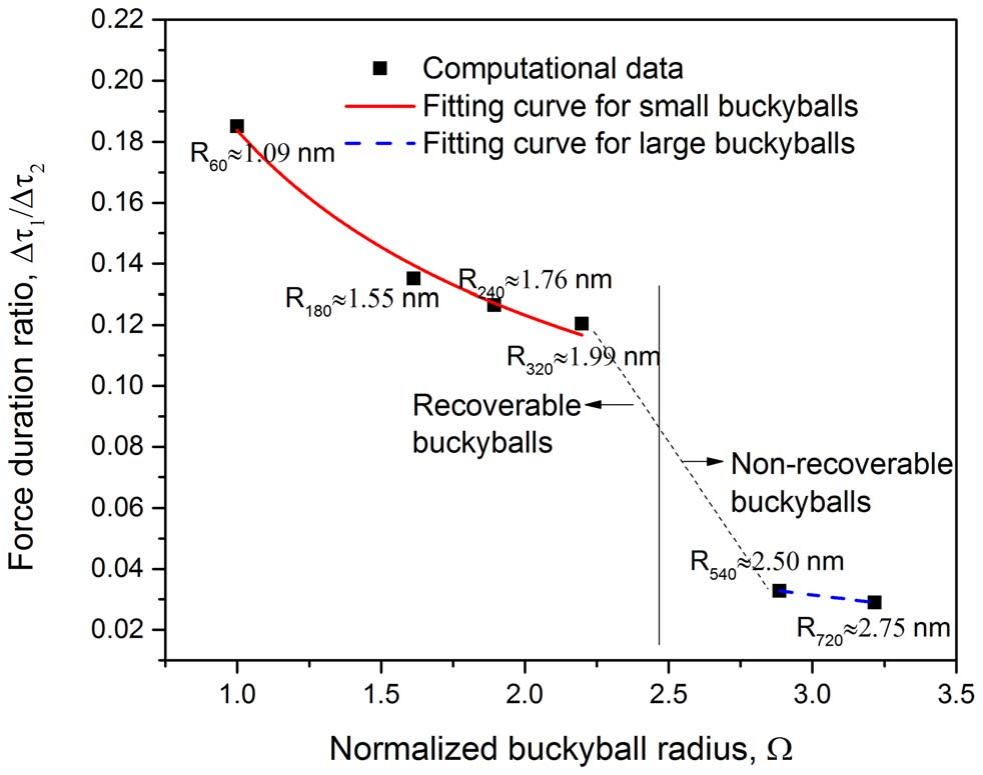
The impulse duration ratios 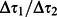 between the impactor and receiver for various buckyballs including C_60_, C_180_, C_240_, C_320_, C_540_ and C_720_ by normalized buckyball radii 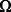 at the impact speed of 500 m/s with the same 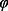 value in each buckyball.

The ratio between the initial and transmitted impulse duration, 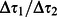, is also an important indicator for energy mitigation. The buckling forces for larger size buckyballs are smaller, owing to the buckling phenomenon. Figure 7 shows the relation between 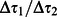 and normalized buckyball diameter 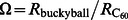 at the impact speed of 500 m/s with the same impactor mass per carbon atom. The sizes of all buckyball involved here are labeled in Figure 7. The 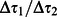 values decay in a power-law manner as the buckyball size increases. More importantly, a sudden drop is observed between C_320_ and C_540_ where the non-recovery phenomenon starts to appear. Once the buckyballs stay in a buckled morphology, the layered and densified structure would create more barriers to transmit the stress waves and the waves are attenuated through the wave reflection among interfaces of buckled shapes. The numerical results may be fitted as
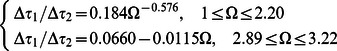
(13)


### Effect of buckyball number

**Figure 8:**
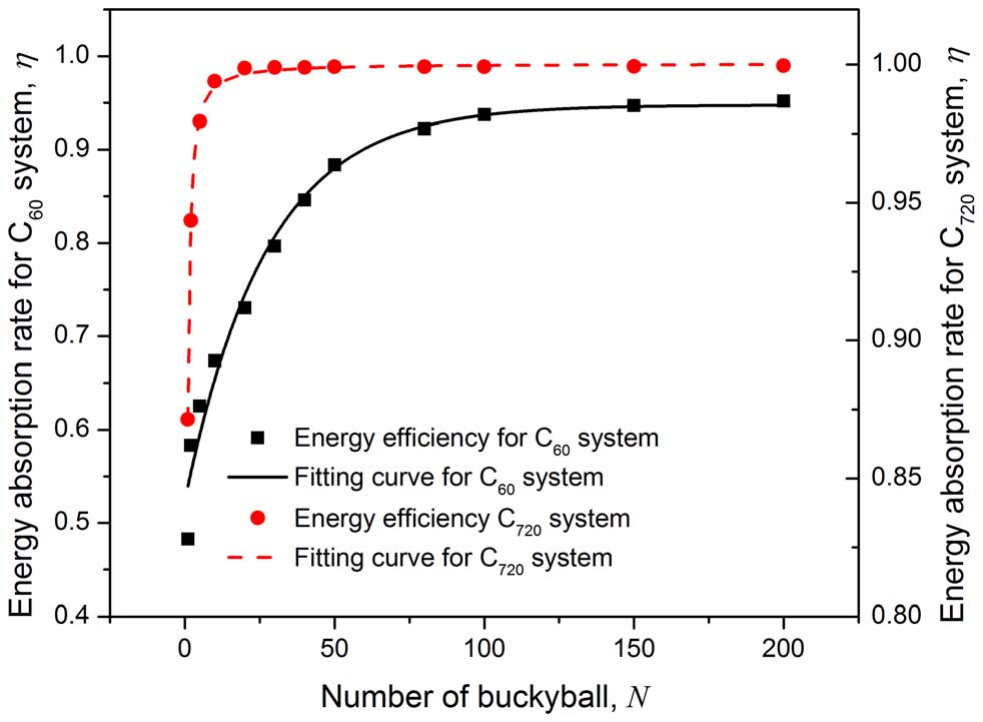
Relations between energy absorption rate and buckyball number for both C_60_ and C_720_ systems at the impact speed 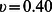 for C_60_ chain system and 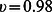 for C_720_ chain system, and the impact mass of 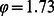 for both systems.

As aforementioned, with the change of buckyball number within the protective system, stress wave propagation characteristics as well as the equivalent system rigidity alters, which may influence the energy mitigation ability of the system for both C_60_ and C_720_ systems. The energy mitigation rate 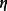 is calculated for systems with buckyball numbers varying from 1 to 200 under the specific impact condition. In Figure 8, one may clearly observe that nonlinear increase on 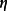 with the buckyball number for both systems. The increasing rate becomes much milder in longer buckyball chains, indicating that there may be a certain length threshold beyond which the system acquires high-efficiency impact wave mitigation. In addition, to reach the same mitigation ability, fewer buckyballs are needed to for larger particles; for example, the system with about 20 C_720_ buckyballs may mitigate over 99% of the impactor kinetic energy (i.e. 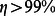), whereas it would take about 80 C_60_ buckyballs to reach 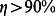, showing another superiority of C_720_ system without the system mass and volume constrain in application.

From systematic simulations, one may also summarize an empirical law at the impact speed 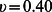 for C_60_ chain system and 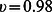 for C_720_ chain system, and the impact mass of 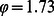 for both systems to describe the relation between buckyball number *N* (*N*>0) and 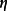 as
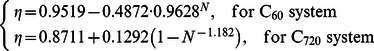
(14)and Eq. (14) may serve as a guidance for engineering design.

## Concluding Remarks

In this paper, the impact mitigation characteristics of a long one dimensional buckyball chain are investigated, which can be extended to granular buckyballs of simple cubic packing. Representative small and large buckyballs, i.e. C_60_ and C_720_ under high speed impact loadings are studied. The impact energy, size and number of buckyballs, are varied in a systematic manner. With relatively small elastic deformations of C_60_ buckyballs during impact, a mechanical model based on Hertz contact law is proposed, with critical parameters calibrated via MD simulations for given impact loading conditions. Energy mitigation is illustrated through force impulse history difference between the impact and receiver. The stress wave propagation speed, the reduction of peak impulse force, and the impulse duration ratio are studied to reveal the dynamic response of the system. The major energy dissipation mechanism for the buckyball chain is the wave reflection among the deformation layers, covalent potential energy, van der Waals interactions as well as the atomistic kinetic energy. These terms may have higher contribution to energy dissipation in C_720_ system with non-recoverable morphologies. Moreover, Buckyball systems are investigated under various impact speeds and impact masses. The smaller mass and higher impact speed results in a higher impulse force attenuation effect, as well as higher system stiffness and shorter wave propagation time. Over 99% and 90% of impact energy for C_720_ and C_60_ chain systems could be mitigated under particular impact conditions respectively and thus a promising buckyball based stress wave mitigation system is suggested. The results may shed lights on the research and development of novel impact/blast protection system.
